# Environmental footprints of Chinese foods and beverages: Literature-based construction of a LCA database

**DOI:** 10.1016/j.dib.2022.108244

**Published:** 2022-05-06

**Authors:** Hongyi Cai, Sander Biesbroek, Xin Wen, Shenggen Fan, Pieter van ’t Veer, Elise F. Talsma

**Affiliations:** a College of Food Science and Nutritional Engineering, China Agricultural University, Beijing, 100083, China; b Division of Human Nutrition and Health, Wageningen University, Stippeneng 4, 6708 WE Wageningen, The Netherlands; c Academy of Global Food Economics and Policy, China Agricultural University, Beijing, 100083, China; d College of Economics and Management, China Agricultural University, Beijing 100083, China

**Keywords:** Greenhouse gas emission, Land use, Water use, Life cycle assessment, Chinese food and drink, Diet

## Abstract

To accurately estimate and model the impact of food consumption and potential dietary changes on environment and climate change, the need for country specific data is evident. This study developed a Chinese Food Life Cycle Assessment Database (CFLCAD) in which Greenhouse Gas Emissions (GHGE) for 80 food items, Water Use (WU) for 93 food items and Land Use (LU) for 50 food items were collected through a literature review. To estimate the environmental footprints of food from production to consumption, the study applied conversion factors for the edible portion of food, food loss ratio and processing, storage, packaging, transportation, and food preparation stages. In addition, when no LCA data of a certain food was available, data from food groups with similar nutritional composition or cultivation condition were used as proxies. The database covered 17 food groups and each food item was referenced to the Chinese Food Composition Table and has a unique food code. The CFLCAD can be used to link individual-level food consumption data with nutrition survey in China, to allow for a more accurate estimation of the environmental footprints of Chinese diets.

## Specifications Table

**Table:** 

Subject	Environmental Engineering
Specific subject area	Diet-related environmental sustainability
Type of data	Figures and tables
How the data were acquired	Data on the environmental footprints of all life cycle stages of food items have been extracted from literature and compiled into Microsoft Excel.
Data format	Analysed data and descriptive statistics
Description of data collection	• Data on the environmental footprints by means of life cycle assessment of food were collected through a literature review in the Chinese National Knowledge Infrastructure (CNKI) and Google Scholar. • Articles and reports written in English or Chinese and published in the years 2005-2020 were identified. • The types of environmental footprints included were Greenhouse gas emission (GHGE), Water use (WU) and Land use (LU). • Articles were excluded if: studies are not available in English or Chinese, or no system boundaries were considered.
Data source location	Food items included in the Chinese Food Life Cycle Assessment Database were based on the Chinese Food Composition Table, resulting in 17 food groups and each food items coded with a unique food code.
Data accessibility	Estimates of environmental footprints of food are available on a data repository with the followinghttps://data.mendeley.com/datasets/37jnjbt454/3.There are three sheets in the excel file which collect data for GHGE, WU and LU. In each sheet, references to data on the environmental impact of food, the year of the study, data type, LCA method, the food code, the food item and the food group are recorded separately.Contact point for further use is Xin Wen at the College of Food Science and Nutritional Engineering, China Agricultural University (wenxin77@cau.edu.cn). Reproduction and translation for non-commercial purposes are authorised, provided the source is acknowledged and the publisher is given prior notice and sent a copy.

## Value of the Data

• This database contains environmental footprint indicators for GHGE, WU and LU of 17 food groups commonly consumed in China.
• The database can be linked to the population dietary intake data to calculate the environmental footprints of individual level food consumption.
• With this dataset a comprehensive assessment of the sustainability of Chinese diets can be done, by including dietary quality, consumer dietary preference choices, and affordability of diets.

## 1 Data Description

**Fig. 1:**
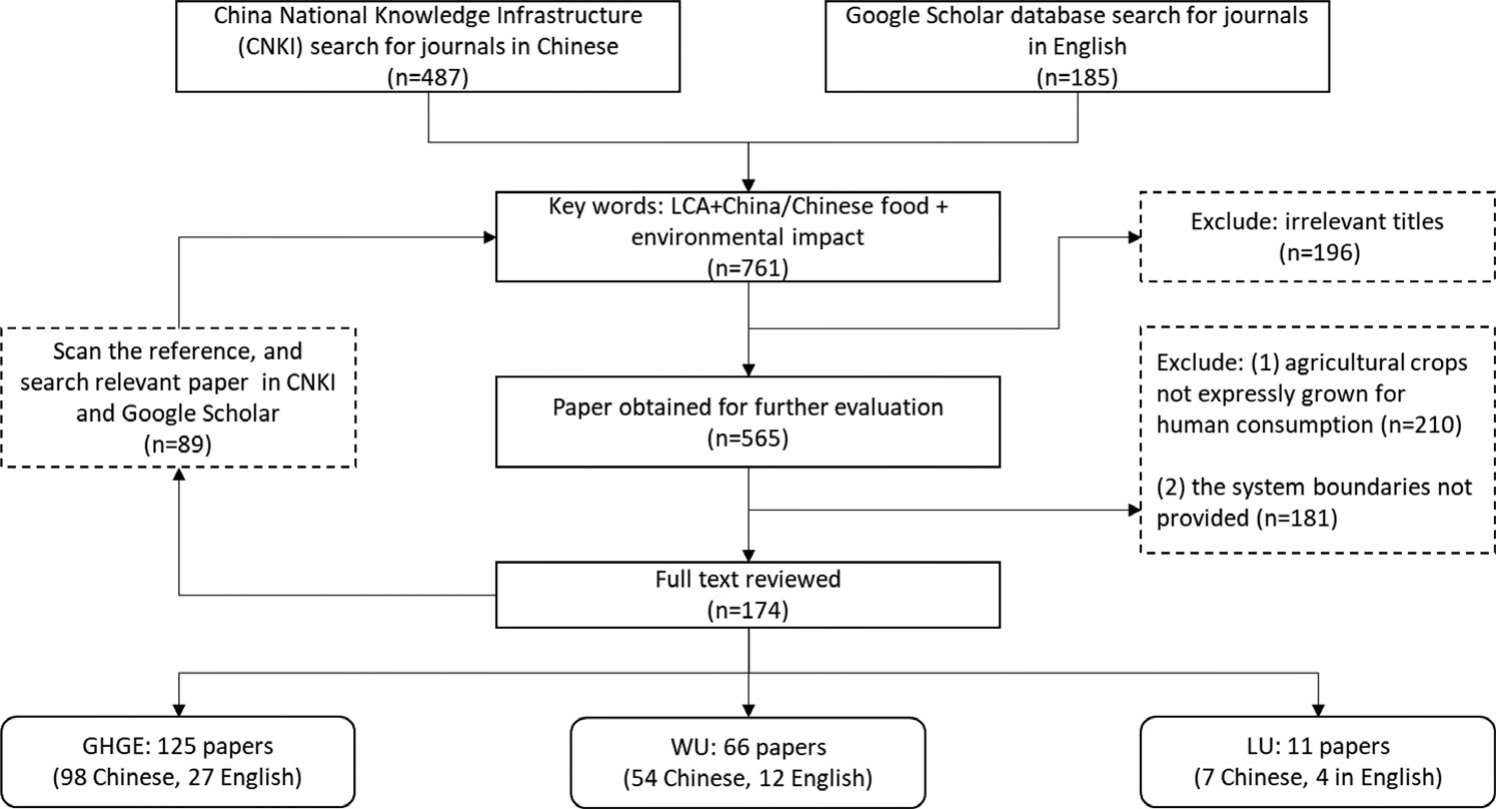
literature review process

The Chinese Food Life Cycle Assessment Database (CFLCAD) provides for each single food item an estimate on Greenhouse gas emissions (GHGE), Water Use (WU) and Land Use (LU) per kg of food as consumed. The food groups in CFLCAD are based on the Chinese Food Composition Table [1], and each food item has a unique food code. Fig. 1 shows the literature search strategy of this study. Table 1 provides summary statistics for GHGE (kg CO_2_-eq/kg food as consumed), WU (m^3^/kg food as consumed), and LU (m^2^/kg food as consumed) from literature for the different food groups in CFLCAD. For this study the GHGE values found in literature were converted to the system boundary off cradle to the post farm gate, and includes the production, storage, processing, packaging, transportation, and preparation at home stages. Table 2 illustrates the GHGE conversion parameters for food groups, and the references of parameters in subsequent post farm gate stages are shown in Appendix Table 1. Table 3 shows the proportion of losses for food groups along the whole food supply chain, and the references of loss proportion are shown in Appendix Table 4. The life cycle inventory data source that was utilized to calculate the environmental footprints for each food group can be found in Table 4. Processed foods and mixed dishes were disaggregated into their basic components and cooked food portions were translated into raw quantities. Table 5 shows the conversion factors for the environmental footprint of food groups of boneless weight of animal-based food. Appendix Table 2 shows 2016 Chinese domestic food production and imports. Appendix Table 3 shows the references of GHGE of food during the package stage. Appendix Table 5 and Table 6 illustrate the calculation of the GHGE of high-frequency consumed processed foods and recipes in China, respectively.

**Table 1:** Environmental footprints values from literature for food groups in the CFLCAD

	Greenhouse gas emissions (GHGE)					Water Use (WU)					Land Use (LU)				
Food group	# Food items	# LCA studies	# GHGE values	Mean (kg CO_2_-eq/kg)	Stdev	# Food items	# LCA studies	# WU values	Mean (m^3^/kg)	Stdev	# Food items	# LCA studies	# LU values	Mean (m^2^/kg)	Stdev
Vegetables	20	22	133	0.266	0.292	23	26	111	0.491	0.775	4	4	8	0.402	0.552
Cereals	15	60	490	1.016	0.806	8	39	468	1.290	0.856	8	6	33	1.538	0.950
Fast foods	2	2	3	1.334	1.616	2	2	3	0.813	0.076	4	1	3	1.920	1.106
Aquatic products	6	10	16	7.029	6.358	17	16	41	3.235	1.881	5	1	10	2.356	2.317
Fruits	9	18	64	0.353	0.246	8	18	53	0.574	0.445	1	1	1	0.640	–
Legumes	4	7	14	0.832	0.681	4	15	49	2.512	0.944	4	1	1	0.810	–
Meat	4	23	122	5.134	2.350	7	28	61	8.970	6.204	8	3	12	13.179	10.197
Sugars and preserves	2	3	4	0.689	0.479	1	6	7	0.797	0.559	3	1	2	1.615	0.955
Beverages	4	3	4	0.931	0.815	3	3	4	5.228	4.735	1	1	1	1.480	
Liquor and alcohol	2	3	4	0.726	0.454	2	6	9	0.803	0.998	2	1	2	1.075	1.223
Poultry	2	11	21	3.784	2.128	5	15	19	3.030	1.105	4	1	4	2.035	0.595
Dairy	3	21	67	1.297	0.404	1	13	14	1.609	0.614	4	2	12	2.911	2.945
Eggs	1	13	22	2.890	1.215	1	15	17	3.257	0.176	1	1	2	1.360	0.156
Nuts and seeds	1	2	2	0.695	0.290	2	7	23	1.400	0.345	–	–	–	–	–
Tubers, starches	3	6	7	0.291	0.367	4	13	41	0.926	0.516	–	–	–	–	–
Fungi and algae	1	1	1	0.930	–	1	1	1	0.270	–	–	–	–	–	–
Fats and oils	1	3	5	1.822	1.404	3	12	19	4.475	1.626	1	1	3	5.210	0.292
**Total**	**80**	**208**	**979**			**93**	**235**	**940**			**50**	**17**	**94**

**Table 2:** GHGE parameters of food groups in subsequent post farm gate stages (kg CO2-eq/kg as produced)*

Food type	Processing	Storage	Transportation	Package	Preparation at home	Total
**Vegetables and fungi**	—	0.005	0.040	0.023	0.005	0.081
**Cereals**	0.007	0.005	0.040	0.023	0.109	0.184
**Fruits and nuts**	—	0.004	0.040	0.023	0.0003	0.075
**Legumes**	0.156	0.005	0.040	0.023	0.006	0.230
**Tubers, starches**	—	0.002	0.025	—	0.005	0.032
**Aquatic products**	—	0.026	0.010	0.023	0.082	0.350
**Meat**	—	0.015	0.087	0.023	0.175	0.603
**Dairy**	0.045	0.015	0.087	0.023	0.016	0.186
**Poultry**	—	0.015	0.044	0.023	0.136	0.521
**Eggs**	—	0.015	0.087	0.023	0.055	0.180
**Beverages**	—	0.002	0.022	0.064	—	0.049
**Sugars and preserves**	0.133	0.005	0.040	0.023	0.005	0.081
**Liquor and alcohol**	—	0.002	0.022	0.064	—	0.049
**Fats and oils**	0.034	—	0.040	—	0.654	0.728

⁎ A version of Table 2 with references is available in the Appendix.

**Table 3:** Loss proportion of food groups in the food supply chain*^,^^1^

Food group	Production	Postharvest handling	Storage	Processing	Transportation	Total
**Vegetables and fungi**	12.15%	19.40%	15.00%	–	5.13%	51.67%
**Cereals**						
**Rice**	3.47%	2.66%	6.17%	2.18%	0.74%	15.22%
**Wheat**	3.12%	0.77%	6.91%	2.38%	0.24%	13.42%
**Corn**	2.17%	1.12%	6.49%	2.27%	0.19%	12.23%
**Fruits and nuts**	9.58%	0.92%	5.36%	–	5.50%	21.36%
**Legumes**	6.00%	3.00%	–	5.00%	1.00%	15.00%
**Tubers, starches**	4.41%	–	17.13%	0.04%	0.01%	21.59%
**Aquatic products**	2.00%	–	4.00%	4.00%	3.20%	13.2%
**Meat**						
**Pork**	11.00%	2.33%	0.89%	0.40%	0.24%	14.86%
**Beef**	10.18%	4.45%	1.04%	0.40%	0.86%	16.93%
**Mutton**	4.15%	2.28%	0.35%	0.40%	0.83%	8.01%
**Dairy**	3.50%	1.00%	–	1.20%	0.50%	6.20%
**Poultry**	8.75%	2.86%	3.24%	0.40%	0.62%	15.87%
**Eggs**	–	–	–	–	–	10.5%
**Beverages**	–	–	–	–	–	5.00%
**Sugars and preserves**	12.15%	19.40%	15.00%	–	5.13%	51.67%
**Liquor and alcohol**	–	–	–	–	–	5.00%
**Fats and oils**	6.00%	3.00%	–	5.00%	1.00%	15.00%

⁎ The dash means that we did not find a relevant coefficient in the literature and therefore the total food loss proportion is underestimated.

1 A version of Table 2 with references is available in the Appendix.

**Table 4:** Number of food items for which LCA data were estimated via different procedures

	Number of food item in CFLCAD withGHGE data					Number of food item in CFLCAD with WU data				Number of food item in CFLCAD withLU data			
Food groups	From literature	Via direct mapping^1^	Via processing^2^	Via recipes^3^	Total	From literature	Via direct mapping^1^	Via recipes^3^	Total	From literature	Via direct mapping^1^	Via recipes^3^	Total
Vegetables	20	181	–	–	201	25	240	–	265	4	133	–	137
Cereals	15	76	2	–	93	12	78	–	91	8	76	–	84
Fruits	9	61	–	–	70	9	75	–	84	1	22	–	23
Legumes	4	64	1	–	69	5	63	–	69	4	66	–	70
Tubers, starches	3	22	–	–	25	4	21	–	25	0	0	–	0
Nuts and seeds	1	40	–	–	41	3	37	–	40	0	0	–	0
Fungi and algae	1	3	–	–	4	1	1	–	2	0	0	–	0
Aquatic products	6	108	–	–	114	30	78	–	108	5	95	–	100
Meat	4	126	–	–	130	8	123	–	131	8	122	–	130
Dairy	3	44	1	–	48	1	38	–	40	4	44	–	49
Poultry	2	40	–	–	42	6	42	–	48	4	22	–	26
Eggs	1	22	–	–	23	1	22	–	23	1	22	–	23
Beverages	4	47	–	–	51	7	35	–	42	1	28	–	29
Fast foods	2	103	2	7	114	3	100	6	112	4	97	6	108
Sugars and preserves	2	21	–	–	23	2	21	–	23	3	20	–	23
Liquor and alcohol	2	44	–	–	46	3	49	–	52	2	44	–	46
Fats and oils	1	6	–	–	7	4	3	–	7	1	6	–	7
Total	80	1008	6	7	1101^4^	124	1026	6	1156^4^	50	797	6	853^4^

1 The environmental impact value was directly mapping to the same food irrespective of the form (i.e., raw, boiled, dried, steamed, or graded, branded).

2 The GHGE for processed foods was calculated by reference to the processing factors in Table 2.

3 Recipe foods were disaggregated into basic components and cooked food portions were translated into raw quantities, and recipes were taken from the Chinese Food Composition Table or the first hit on internet.

4 The total number of the three indicators varies due to the different amounts of literature on the GHGE, WU and LU of food.

**Table 5:** The conversion ratios of boneless weight of animal-based food

	Sheep	Chicken	Beef	Pork	Fish
Ratio boneless weight: live weight	33% [16,17]	65% [18]	46% [19]	43% [20]	54% [1]
Ratio boneless weight: carcass weight	67% [16,17]	75% [18]	83% [19]	62% [20]	–

## 2 Experimental Design, Materials and Methods

### 2.1 Literature review

#### 2.1.1 Search strategy and data sources

The CFLCAD was developed based on a literature review using the China National Knowledge Infrastructure (CNKI) for journals in Chinese and Google Scholar databases for journals in English. The searching keywords were “LCA” or “life cycle assessment”, “China or Chinese”, “food” and “food name”. Studies were selected when any of the types of environmental footprint namely GHGE, WU, and LU was reported and when the articles and reports were published between 2005 to 2020. We realized that potential biases exist in studies with different life cycle assessment methods. One study compared LCA studies that used economic-input output (EIO) modelling (top-down studies) to studies that used process-based modelling (bottom-up studies) and showed a strong correlation exists between methodological choices [2]. Furthermore, several studies analyzed impacts on global warming, energy demand and consumptive water use from meat processing of chicken, pork, sheep and beef. They compared LCA methodologies between process based, EIO, and hybrid methodologies [3], with results generally remaining within a similar range. Therefore, we can accept errors within a certain range through the method of literature review. The literature review strategy is shown in Fig. 1.

#### 2.1.2 Inclusion and exclusion of literature

Articles were included when the system boundary of the LCA studies includes at least “cradle to farm-gate” and when the functional unit of GHGE, WU and LU values were reported in kg CO_2_-eq/kg, m^3^/kg, and m^2^/kg, respectively. Articles were excluded when the agricultural crops studied were not grown for human consumption (e.g., for biofuels, timber, fibers, cotton) or when the system boundary was not specified. If review articles were retrieved in the literature search, the reference list was scanned and used for identify the original LCA papers. After full text screening, for GHGE, this resulted in a total of 125 papers, of which 98 in Chinese and 27 in English. For WU, this resulted in a total of 66 papers, of which 54 in Chinese and 12 in English. For LU, this resulted in a total of 11 papers, 7 in Chinese and 4 in English. The average values of GHGE, WU and LU from literature are presented in Table 1.

### 2.2 Environmental footprints from production to consumption

Most LCA studies used the farm gate or production phase as system boundaries and excluded the preparation, consumption, and waste management phases. Especially, for GHGE, this results in an underestimation of the actual environmental footprints of food products. To resolve the data gap, the environmental footprints values in this study were converted to the system boundary from cradle to the post farm gate, by using conversion factors on production, storage, processing, packaging, transportation, preparation at home stages, as well as the food losses along the food supply chain from literature. The appropriate conversion parameters were acquired from literature data and statistical yearbooks to calculate the environmental footprints of the post farm gate stage. It was found that no significant increases in WU and LU were detected in the post-farm gate phase [4], [5], [6]. For the system boundaries of WU and LU, this study did not include the storage, transportation, packaging, and preparation at home stages. GHGE conversion parameters of food groups in each post farm gate stage are shown in Table 2, and the calculation of post-farm gate of GHGE are shown in Section 2.2.1 to 2.2.4. Furthermore, for all three environmental footprints indicators, pot-farm gate losses were considered and shown in Section 2.2.5.

#### 2.2.1 GHGE of different food groups during the processing stage

For cereals, vegetable oils and pulses, processing is concerned with primary processing of agricultural by-products. Grain was assumed to be processed by medium-sized grain milling machine, with main parameters including capacity of 4.5 t/h, and power of 41 kW. The main parameters of vegetable oil processing machinery were assumed as capacity of 210 kg/h, power of 7.5 kW. Soybean was assumed to be mainly processed to tofu by machinery with capacity of 30 kg/h and power of 5.5 kW [7]. Table 2 shows the GHGE per unit mass of energy consumed in the processing. Dairy products need cooling and sterilization before selling as foods and beverages and therefore the GHGE parameters of the work of Gan et al. (2019) for dairy processing were applied [8]. The calculation of GHGE for processed foods were derived from different sources and can be found in Appendix Table 1.

#### 2.2.2 GHGE of different food groups during the storage stage

In the storage stage, the distribution centre is normally equipped with large-scale cold storage and other refrigeration facilities to ensure that the fresh food remains fresh before distribution [9,10]. This refrigeration system requires a large amount of energy and therefore the storage volume of food and the storage time of the food in the cold storage are the main factors affecting GHGE. The GHGE of food products during the storage stage were shown in Table 2, and the parameters were derived from different sources and can be found in Appendix Table 1.

#### 2.2.2 GHGE of different food groups during the transportation stage

GHGE of food during the transportation stage includes energy used by refrigerating agents and vehicles in the transportation process (international and national). At the time of this study, 90% of the food consumed in China was produced domestically (Appendix Table 2). For two food items these were not the case, i.e., barley and oil crops, of which more than 50% of the available food was imported. However, because these food items comprised a small amount of the total diet by weight, GHGE, WU, and LU were quantified using China-specific production data. For national transportation, transport distances by truck to wholesalers and retailers were assumed to be 400 km and 100 km, respectively [11, 12]. The GHGE of food products during the transportation stage were shown in Table 2, and the parameters from different sources can be found in Appendix Table 1.

#### 2.2.3 GHGE of different food groups during the package stage

The GHGE of food during packaging stage were obtained from the research results of Kuai et al. (2013) [13]. Kuai et al. (2013) conducted research on GHGE of five shopping bags commonly used by Chinese consumers, namely high-density polyethylene (HDPE) plastic bags, low-density polyethylene (LDPE) plastic bags, paper shopping bags, non-woven shopping bags and cotton shopping bags. The specifications of the five types of shopping bags are shown in Appendix Table 3*,* and the parameters can be found in Appendix Table 1.

#### 2.2.4 GHGE of different food groups during preparation at home stage

The GHGE of preparation at home stage were derived from Huang et al. (2021) [14] (Appendix Table 1). For vegetables and legumes we assumed they needed to be cooked for 2 minutes per 500 grams, meat for 40 minutes per 500 grams, aquatic products for 20 minutes per 1 kilogram, eggs for 10 minutes per 200 grams, and poultry for 20 minutes per 1 kilogram. At present, most residents in China use natural gas for cooking, and the average consumption of natural gas is 0.4 m^3^/h. The electricity consumption for rice cooking was calculated assuming that for 500 grams or rice, a rice cooker of 900W would take 35 minutes.

#### 2.2.5 Food loss proportion of food groups

Food losses are an important factor in estimating environmental footprints of diets as foods produced but not consumed also contribute to the overall system impact. Food losses in this study included losses during storage, processing, packaging, transportation, retailing, and preparation at home. Percentages of food losses were estimated at the level of food groups. Table 3 shows the food loss proportions in the whole food chain of the food that is frequently consumed in China based on weight that were used to calculate the GHGE, WU and LU. The food loss proportions were derived from different sources and can be found in Appendix Table 4.

### 2.3 Matching environmental footprints of food groups to the Chinese Food Composition Table

#### 2.3.1 Matching to the single food items

The environmental footprints value was directly assigned to the same food irrespective of the form (i.e., raw, boiled, dried, steamed, or graded, branded). For example, GHGE value for “wheat flour” was assigned as same to both “wheat flour, refined, special grade 1” and “wheat flour, refined, special grade 2”. When no LCA data of a certain food was available, data from similar food groups were used as proxies (Table 4). Data on land use for nuts, fungi, and tubers were not found in our literature search. However, these food groups comprise a very small amount of the total diet by weight and therefore fungi and tubers were based on the average of vegetables, while values for nuts were based on fruits based on similarity of cultivation condition.

#### 2.3.2 Matching to the recipe

For recipes, a break-down into ingredients was needed before linking these to their corresponding primary food items. To integrate the dietary intake data with the GHGE data, processed foods and mixed dishes were disaggregated into their basic components and cooked food portions were translated into raw quantities. Furthermore, recipes taken from the Chinese Food Composition Table were used to break down composite foods into their ingredients, but if recipes from food composition table were not available, the first hit on internet was used. All recipes for composite foods were assumed to be homogenous across China (Appendix Table 5 & 6). Food groups of CFLCAD and their corresponding life cycle inventory data source used for quantifying environmental footprints are shown in Table 4.

#### 2.3.3 Conversion to edible portion

For the edible part of the food, the environmental footprints for animal-base foods were converted to a common functional unit in per kg boneless weight. The conversion ratios were derived from the literature as shown in Table 5. In LCA studies of plant-based foods, the weight basis is the form in which it is delivered or purchased (e.g., whole apples, bananas with peels) [15]. To reconcile this inconsistency, the conversion factors of edible portion were drawn from the Chinese Food Composition Table.

#### 2.4 Calculations of the tabulated values of GHGE, WU and LU

The total GHGE, WU and LU per kg of food as consumed were calculated using the following formula, respectively:

- Total GHGE = GHGE from cradle to farm gate * (1/edible portion parameter) * (1/losses and waste parameter) + GHGE during storage + GHGE during transportation + GHGE during packaging + GHGE during preparation at home
- Total WU = WU from cradle to farm gate * (1/edible portion parameter) * (1/losses and waste parameter)
- Total LU = LU from cradle to farm gate * (1/edible portion parameter) * (1/losses and waste parameter)

## Declaration of Competing Interest

The authors declare that they have no known competing financial interests or personal relationships that could have appeared to influence the work reported in this paper.

## Data Availability

Chinese Food Life Cycle Assessment Database (Original data) (Mendeley Data).

## Appendix Supplementary materials

Image, application 1
