# Supplementary material for: Environmental footprints of Chinese foods and beverages: Literature-based construction of a LCA database
Source: Data Brief. 2022 May 6;42:108244. doi: 10.1016/j.dib.2022.108244 (PMC9117526; doi:10.1016/j.dib.2022.108244)
Supplement: Supplementary file 1 [file mmc1.docx]

**Environmental footprints of Chinese foods and beverages: Literature-based construction of an LCA database**

Hongyi Cai ^a^, [Sander Biesbroek](https://sciprofiles.com/profile/980707) ^a,^ ^[[1]](#footnote-1)^*, Xin Wen ^b^, Shenggen Fan ^c, d^, [Pieter van’t Veer](https://sciprofiles.com/profile/author/dW1FRjZXRzdwMnlWb25mMW1UZ2NESkFvK1VtNEcxMjNOL29KbVlZRUJYYz0=) ^a^, Elise Talsma ^a^

*^a^ Division of Human Nutrition and Health, Wageningen University, Stippeneng 4, 6708 WE Wageningen, The Netherlands*

*^b^ College of Food Science and Nutritional Engineering, China Agricultural University, Beijing, 100083, China*

*^c^ Academy of Global Food Economics and Policy, China Agricultural University, Beijing, 100083, China*

*^d^ College of Economics and Management, China Agricultural University, Beijing 100083, China*

**Appendix- Supplementary Material**

### *Appendix Table 1. GHGE parameters of food groups in subsequent post farm gate stages (kg CO2-eq/kg as produced)*

| **Food type** | **Processing** | **Storage** | **Transportation** | **Package** | **Preparation at home** | **Total** |
| --- | --- | --- | --- | --- | --- | --- |
| **Vegetables and fungi** | — | 0.005[1,2] | 0.040[1,2] | 0.023[3,4] | 0.005[5] | 0.081 |
| **Cereals** | 0.007[6] | 0.005[1,2] | 0.040[1,2] | 0.023[3,4] | 0.109[5] | 0.184 |
| **Fruits and nuts** | — | 0.004[1,2,7] | 0.040[1,2] | 0.023[3,4] | 0.0003[5] | 0.075 |
| **Legumes** | 0.156[6] | 0.005[1,2] | 0.040[1,2] | 0.023[3,4] | 0.006[5] | 0.230 |
| **Tubers, starches** | — | 0.002[8] | 0.025[8] | — | 0.005[5] | 0.032 |
| **Aquatic products** | — | 0.026[9] | 0.010[9] | 0.023[3,4] | 0.082[5] | 0.350 |
| **Meat** | — | 0.015[10] | 0.087[10] | 0.023[3,4] | 0.175[5] | 0.603 |
| **Dairy** | 0.045[11] | 0.015[10] | 0.087[10] | 0.023[3,4] | 0.016[5] | 0.186 |
| **Poultry** | — | 0.015[10] | 0.044[12] | 0.023[3,4] | 0.136[5] | 0.521 |
| **Eggs** | — | 0.015[10] | 0.087[10] | 0.023[3,4] | 0.055[5] | 0.180 |
| **Beverages** | — | 0.002[13–15] | 0.022[13–16] | 0.064[13–15] | — | 0.049 |
| **Sugars and preserves** | 0.133[17] | 0.005[1,2] | 0.040[1,2] | 0.023[3,4] | 0.005[5] | 0.081 |
| **Liquor and alcohol** | — | 0.002[13–15] | 0.022[13–16] | 0.064[13–15] | — | 0.049 |
| **Fats and oils** | 0.034[6] | — | 0.040[1,2] | — | 0.654[5] | 0.728 |

### *Appendix Table 2. Overview of Chinese domestic food production and imports, 2016 (ten thousand tons, %)*

| Food items | Domestic net production | Total import | Domestic net production (%) | Total import (%) |
| --- | --- | --- | --- | --- |
| Cereals | 48399.6 | 2167.1 | 95.7% | 4.3% |
| Wheat and products | 12121.8 | 757.2 | 94.1% | 5.9% |
| Rice and products | 13630.8 | 271.4 | 98.0% | 2.0% |
| Barley and products | 108.4 | 252.8 | 30.0% | 70.0% |
| Corn and products | 21837.2 | 740.7 | 96.7% | 3.3% |
| Sorghum and products | 287.8 | 119.8 | 70.6% | 29.4% |
| Starchy roots | 17234 | 3167.1 | 84.5% | 15.5% |
| Sugar crops | 13810.6 | 92.9 | 99.3% | 0.7% |
| Sugar and sweetener | 1506.6 | 569.4 | 72.6% | 27.4% |
| Beans (excluding soybeans) | 364.1 | 118.1 | 75.5% | 24.5% |
| Tree nuts | 324.8 | 58.1 | 84.8% | 15.2% |
| Oil crops | 5709.9 | 7037.1 | 44.8% | 55.2% |
| Soybeans | 1167.4 | 6556.4 | 15.1% | 84.9% |
| Vegetable oil | 2175.7 | 1167.3 | 65.1% | 34.9% |
| Palm oil | 15.8 | 709.8 | 2.2% | 97.8% |
| Vegetables | 57040.9 | 167.4 | 99.7% | 0.3% |
| Fruits | 14638.4 | 570 | 96.3% | 3.7% |
| Fast food | 146.9 | 43.8 | 77.0% | 23.0% |
| Spices | 43.8 | 2.8 | 94.0% | 6.0% |
| Alcoholic beverages | 6512.3 | 115.4 | 98.3% | 1.7% |
| Meat | 8322.1 | 442.2 | 95.0% | 5.0% |
| Animal fat | 401.5 | 66.3 | 85.8% | 14.2% |
| Eggs | 2902.7 | 12.6 | 99.6% | 0.4% |
| Milk (without butter) | 4033.7 | 978 | 80.5% | 19.5% |
| Fish and sea food | 6358.2 | 1126.7 | 84.9% | 15.1% |

*Data source: FAOSTAT*[18]

### *Appendix Table 3. Specifications and GHGE of five shopping bags*

|  | HDPE plastic bags | LDPE plastic bags | Paper bags | Non-woven bags | Cotton bags |
| --- | --- | --- | --- | --- | --- |
| Volume(L) | 18.1 | 17.8 | 18.2 | 18 | 18.5 |
| Length*width*height (cm) | 37*12*40 | 33*13*42 | 35*13*40 | 40*10*45 | 35*12*44 |
| Single bag weight (g) | 5.68 | 10.45 | 23.8 | 60.0 | 130.0 |
| GHGE (kg CO_2_-eq/kg) | 0.023 | 0.034 | 0.092 | 0.31 | 3.12 |

*Data source: Kuai, et al. (2013) [3], Luo, et al. (2021) [4]*

### *Appendix Table 4. Loss proportion of food groups in the food supply chain**

| **Food group** | **Production** | **Postharvest handling** | **Storage** | **Processing** | **Transportation** | **Total** |
| --- | --- | --- | --- | --- | --- | --- |
| **Vegetables and fungi** | 12.15%[19–23] | 19.40%[19,20] | 15.00%[19,20] | -- | 5.13%[19,20] | 51.67%[19,20] |
| **Cereals** |  |  |  |  |  |  |
| **Rice** | 3.47%[19–23] | 2.66%[19–23] | 6.17%[19–23] | 2.18%[19–23] | 0.74%[19–23] | 15.22%[19–23] |
| **Wheat** | 3.12%[19–23] | 0.77%[19–23] | 6.91%[19–23] | 2.38%[19–23] | 0.24%[19–23] | 13.42%[19–23] |
| **Corn** | 2.17%[19–23] | 1.12%[19–23] | 6.49%[19–23] | 2.27%[19–23] | 0.19%[19–23] | 12.23%[19–23] |
| **Fruits and nuts** | 9.58%[19–23] | 0.92%[19–23] | 5.36%[19–23] | -- | 5.50%[19–23] | 21.36%[19–23] |
| **Legumes** | 6.00%[19] | 3.00%[19] | -- | 5.00%[19] | 1.00%[19] | 15.00%[19] |
| **Tubers, starches** | 4.41%[19] | -- | 17.13%[19] | 0.04%[19] | 0.01%[19] | 21.59%[19] |
| **Aquatic products** | 2.00%[19,24] | -- | 4.00%[19,24] | 4.00%[19,24] | 3.20%[19,24] | 13.2%[19,24] |
| **Meat** |  |  |  |  |  |  |
| **Pork** | 11.00%[19,24] | 2.33%[19,24] | 0.89%[19,24] | 0.40%[19,24] | 0.24%[19,24] | 14.86%[19,24] |
| **Beef** | 10.18%[19,24] | 4.45%[19,24] | 1.04%[19,24] | 0.40%[19,24] | 0.86%[19,24] | 16.93%[19,24] |
| **Mutton** | 4.15%[19,24] | 2.28%[19,24] | 0.35%[19,24] | 0.40%[19,24] | 0.83%[19,24] | 8.01%[19,24] |
| **Dairy** | 3.50%[19] | 1.00%[19] | -- | 1.20%[19] | 0.50%[19] | 6.20%[19] |
| **Poultry** | 8.75%[19,24] | 2.86%[19,24] | 3.24%[19,24] | 0.40%[19,24] | 0.62%[19,24] | 15.87%[19,24] |
| **Eggs** | -- | -- | -- | -- | -- | 10.5%[25] |
| **Beverages** | -- | -- | -- | -- | -- | 5.00%[14] |
| **Sugars and preserves** | 12.15%[19,20] | 19.40%[19,20] | 15.00%[19,20] | -- | 5.13%[19,20] | 51.67%[19,20] |
| **Liquor and alcohol** | -- | -- | -- | -- | -- | 5.00%[14] |
| **Fats and oils** | 6.00%[19] | 3.00%[19] | -- | 5.00%[19] | 1.00%[19] | 15.00%[19] |

**The dash means that we did not find a relevant coefficient in the literature and therefore the total food loss proportion is underestimated.*

### *Appendix Table 5. Calculation of the GHGE of high-frequency consumed processed food in the Chinese Food Composition Table based on weight**

| Food groups | | Cereals | | Ethnic foods and cakes | Dried legumes and legume products |
| --- | --- | --- | --- | --- | --- |
| Food items | | Noodle | Rice flour  Flat rice-noodles | Bread | Tofu |
|  |  | Steamed bread |  |  |  |
|  |  | Dried noodles |  |  |  |
|  |  | You Tiao |  |  |  |
|  |  | Pancake |  |  |  |
|  |  | Rice flour |  |  |  |
|  |  | Flat rice-noodles |  |  |  |
| Data sources (kg CO_2_-eq/ kg) | GHGE of agricultural activity | 0.978 | 1.21 | 0.978 | 0.792 |
|  | GHGE of post farm gate | 0.007 | 0.007 | 0.007 | 0.156 |
|  | Total | 0.985 | 1.217 | 0.985 | 0.948 |

*^*^Recipes taken from the Chinese Food Composition Table are used to break-down composite foods into its ingredients, if the food composition table is not available, the ﬁrst hit on internet will be referred.*

### *Appendix Table 6. Calculation of the high-frequency consumed recipe in Chinese Food Composition Table based on weight**

| Food group | Food sub-groups | Food items | Data of GHGE sources (kg CO_2_-eq/ kg) | The proportion of ingredients used in the food recipe table^1^ | Total (kg CO_2_-eq/ kg) |
| --- | --- | --- | --- | --- | --- |
| Ethnic foods and cakes | Convenience food | Steamed Bun (Pork Filling) | Pork (fat and lean) | 62.5% | 2.963 |
|  |  |  | Wheat flour (standard flour) | 37.5% |  |
|  |  | Dumplings (pork and cabbage stuffing) | Pork (fat and lean) | 70% | 2.6852 |
|  |  |  | Cabbage | 10% |  |
|  |  |  | Wheat flour (standard flour) | 20% |  |
|  |  | Biscuits | Egg | 1/3 | 1.552 |
|  |  |  | Wheat flour (standard flour) | 2/3 |  |
|  | Cake | Cake | Egg | 60% | 2.196 |
|  |  |  | Milk | 20% |  |
|  |  |  | Wheat flour (standard flour) | 20% |  |

*^*^Recipes taken from the Chinese Food Composition Table are used to break-down composite foods into its ingredients, if the food composition table is not available, the ﬁrst hit on internet will be referred.*

**Reference**

[1] Runan Xu, Bin Liu, Aiqiang Chen, et al. Analysis of Cold Chain Carbon Footprint of Fruits and Vegetables in China. Journal of Refrigeration. 39 (2018) 13-18+25. (in Chinese)

<http://dx.doi.org/10.18462/iir.iccc.2018.0001>

[2] Bin Li, Bin Liu, Aiqiang Chen, et al. Calculation of Carbon Footprint of Agricultural Products Based on the Model of Cold Chain. Journal of Refrigeration Technology. 42 (2019) 1–5. (in Chinese)

[DOI:](https://www.cnki.com.cn/Article/CJFDTotal-LCJZ201903001.htm) [10.3969/j.issn.1674-0548.2019.03.001](http://dx.chinadoi.cn/10.3969/j.issn.1674-0548.2019.03.001)

[3] Meijuan Kuai, Jinshu Wang, Dong Wang, et al. Analysis on Life Cycle Carbon Footprint of Several Major Kinds of Shopping Bags in Xuzhou City. Ecological Economy, (2013) 49–55. (in Chinese)

<https://www.cnki.com.cn/Article/CJFDTOTAL-STJX201301012.htm>

[4] Ziqian Luo. Study on energy consumption of takeaway food packaging based on life cycle evaluation method. Reform and strategy. 37 (2021) 111–124. (in Chinese)

DOI: [10.16331/j.cnki.issn1002-736x.2021.01.012](http://dx.chinadoi.cn/10.16331/j.cnki.issn1002-736x.2021.01.012)

[5] Heping Huang, Yali Li, Siling Yang. Spatio-Temporal Evolution Characteristics of Carbon Emissions from Food Consumption of Urban Residents in China. Chinese Journal of Environmental Management, 13 (2021) 112–120. (in Chinese)

DOI: [10.16868/j.cnki.1674-6252.2021.01.112](http://dx.chinadoi.cn/10.16868/j.cnki.1674-6252.2021.01.112)

[6] WU Yan, WANG Xiaoke, LU Fei, The carbon footprint of food consumption in Beijing, Acta Ecol. Sin. 32 (2012) 1570–1577. (in Chinese)

DOI:[10.5846/stxb201101140074](http://dx.doi.org/10.5846/stxb201101140074)

[7] Li You. A Research on Cold Chain Logistics System Based on Carbon Footprint. Guangzhou University, MA thesis, 2017. (in Chinese)

<https://kns.cnki.net/KCMS/detail/detail.aspx?dbcode=CMFD&dbname=CMFD201801&filename=1017082871.nh&v=>.

[8] Jiawen Zhang. Calculation of the carbon footprint of agricultural logistics based on the LCA method. Modern Marketing. (2018) 68–69. (in Chinese)

<https://www.cnki.com.cn/Article/CJFDTotal-XIXX201810061.htm>

[9] Y. Dong, S.A. Miller, Assessing the lifecycle greenhouse gas (GHG) emissions of perishable food products delivered by the cold chain in China, J. Clean. Prod. 303 (2021) 126982.

<https://doi.org/10.1016/j.jclepro.2021.126982>

[10] Shuanglin Dong, Fang Lin, Fukai Luo, et al. Calculation of the carbon emission and its comparison with the pollution in the fish-culture enterprise. Chinese Fisheries Economics. 28 (2010) 38–43. (in Chinese)

[DOI:](https://www.cnki.com.cn/Article/CJFDTotal-ZYJJ201006005.htm) [10.3969/j.issn.1009-590X.2010.06.007](http://dx.chinadoi.cn/10.3969/j.issn.1009-590X.2010.06.007)

[11] Yutian Gan. Research on the Estimation and Influence Factors of Carbon Emission of Dairy Cattle Industry in China. Northeast Agricultural University, MA thesis, 2019. (in Chinese)

<https://kns.cnki.net/KCMS/detail/detail.aspx?dbcode=CMFD&dbname=CMFD201902&filename=1019176249.nh&v=>.

[12] Bo Lu. Environmental impact assessment of commercial broiler production based on life cycle. Shenyang Agricultural University, MA thesis, 2020. (in Chinese)

[DOI: 10.27327/d.cnki.gshnu.2020.000441](https://kns.cnki.net/KCMS/detail/detail.aspx?dbcode=CMFD&dbname=CMFD202002&filename=1020979020.nh&v=.)

[13] Hong Li, Wujiu Zhang, Changxin Song, et al. A study of the whole life cycle carbon footprint of beer in China. Beer Tech. (2013) 14-21+13. (in Chinese)

DOI: [10.3969/j.issn.1008-4819.2013.02.006](http://dx.chinadoi.cn/10.3969/j.issn.1008-4819.2013.02.006)

[14] Feng Gao, Zefeng Guo, Research on the carbon footprint of beer production based on life circle assessment. Beer Tech. (2013) 7-10+6. (in Chinese)

DOI: [10.3969/j.issn.1008-4819.2013.03.004](http://dx.chinadoi.cn/10.3969/j.issn.1008-4819.2013.03.004)

[15] Jingjing Yang, Junhui Zhong, Kun Zhang, et al. Beer carbon footprint and a case study. Beer Tech. (2013) 6–14. (in Chinese)

DOI: [10.3969/j.issn.1008-4819.2013.08.004](http://dx.chinadoi.cn/10.3969/j.issn.1008-4819.2013.08.004)

[16] Jingjing Yang, Jian Lu, Junhui Zhong. Analysis of the impact of transport processes on the carbon footprint of beer. Beer Tech. (2014) 24–30. (in Chinese)

[DOI:](https://www.cnki.com.cn/Article/CJFDTOTAL-PJKJ201405008.htm) [10.3969/j.issn.1008-4819.2014.05.008](http://dx.chinadoi.cn/10.3969/j.issn.1008-4819.2014.05.008)

[17] X. Xu, Y. Lan, A comparative study on carbon footprints between plant- and animal-based foods in China, J. Clean. Prod. 112 (2016) 2581–2592.

<https://doi.org/10.1016/j.jclepro.2015.10.059>.

[18] FAOSTAT, (n.d.). http://www.fao.org/faostat/en/ (accessed May 21, 2021).

[19] L. Xue, X. Liu, S. Lu, G. Cheng, Y. Hu, J. Liu, Z. Dou, S. Cheng, G. Liu, China’s food loss and waste embodies increasing environmental impacts, Nat. Food. 2 (2021) 519–528.

<https://doi.org/10.1038/s43016-021-00317-6>.

[20] G. Liu, Food Losses and Food Waste in China: A First Estimate, OECD, Paris, 2014. <https://doi.org/10.1787/5jz5sq5173lq-en>.

[21] Yi Luo, Xuanfu Li, Dong Huang, et al. The progress and prospects of research on food loss and waste. Journal of Natural Resources, 35 (2020) 1030–1042. (in Chinese)

DOI: [10.31497/zrzyxb.20200502](https://doi.org/10.31497/zrzyxb.20200502)

[22] Liwei Gao, Study on the characteristics of supply chain losses and wastage of major food crops in China and their loss reduction potential. Chinese Academy of Agricultural Sciences, 2019. (in Chinese) <https://kns.cnki.net/kcms/detail/detail.aspx?dbcode=CDFD&dbname=CDFDLAST2019&filename=1019054673.nh&uniplatform=NZKPT&v=7W3zi_HrYsa6zjzxYWr2UUa6Kq17RaB93qy_kMKikiWfRHTdk8Ddx2zY8TpahcFx>

[23] Shijun Lu, Xiaojie Liu, Li Xue, et al. Addressing the Losses and Waste of Chinese Rice Supply Chain: Sources, Drivers and Mitigation Strategies. Scientia Agricultura Sinica, 52 (2019) 3134–3144.

DOI: [10.3864/j.issn.0578-1752.2019.18.006](http://dx.chinadoi.cn/10.3864/j.issn.0578-1752.2019.18.006)

[24] Lin Zhou, Zhenni Yang, Min Zhang, et al. Whole-Industry Chain Loss and Edible Rate of Chinese Meats. Scientia Agricultura Sinica, 52 (2019) 3934–3942.

DOI: [10.3864/j.issn.0578-1752.2019.21.020](http://dx.doi.org/10.3864/j.issn.0578-1752.2019.21.020)

[25] Kaiguo Sun, Evaluation study of China's poultry egg distribution model, Capital University of Economics and Business, 2014. (Chinese) <https://kns.cnki.net/KCMS/detail/detail.aspx?dbcode=CMFD&dbname=CMFD201402&filename=1014215283.nh&v=>.

1. * *Corresponding author. E-mail address:* [*sander.biesbroek@wur.nl*](mailto:sander.biesbroek@wur.nl) *(S. Biesbroek)* [↑](#footnote-ref-1)
